# Measuring Social Vulnerability in an Urban Space Through Multivariate Methods and Models

**DOI:** 10.1007/s11205-021-02680-0

**Published:** 2021-04-28

**Authors:** Cristina Davino, Marco Gherghi, Silvia Sorana, Domenico Vistocco

**Affiliations:** 1 Department of Economics and Statistics, University of Naples Federico II, Naples, Italy; 2 Department of Political Sciences, Communications and International Relationships, University of Macerata, Macerata, Italy; 3 Department of Political Science, University of Naples Federico II, Naples, Italy

**Keywords:** Composite indicators, Social vulnerability, Multivariate analysis, Urban space

## Abstract

This article proposes a quantitative analysis to measure social vulnerability in a urban space, specifically in the area of the Municipality of Rome. Social vulnerability can be defined as a situation in which people are characterized by a condition of multidimensional deprivation that encompasses multiple aspects of life and exposes population to different risks and hazards produced by natural, environmental, socioeconomic and epidemic factors. The analysis that follows presents the use of multivariate methods and models to provide an index for every dimensions of vulnerability (housing quality, social context, education, employment, urban mobility, social relations and economic conditions) and a global index for mapping social vulnerability. The analysis also succeeds in identifying significant vulnerability spatial patterns and to measure how and if vulnerability can be different in case of an observed heterogeneity in urban space.

## Introduction

The need to understand the social phenomena linked to urban spaces comes from the fact that the concentration of millions of people in urban areas is very often accompanied by various forms of inequality such as unequal access to resources, services, employment opportunities and training. The configuration of urban spaces assumes a prominent role because it enables individuals to flourish, aspire and be able to escape poverty, illness and other serious forms of deprivation. The shape of the city, its mutation and expansion are the most tangible products of this type of interaction.

This paper aims to assess social vulnerability by identifying its dimensions and analysing its distribution at urban scale. The ultimate goal is the detection of city’s areas most exposed to new social risks.

The proposed quantitative analysis is based on the consideration that vulnerability is a complex concept that cannot be directly operationalised through a single indicator but it is a composite indicator (CI) obtained as a combination of several indicators. In this regard, multidimensional data analysis can support in facing the main issues in the CI construction. In essence, the main challenges of the current state of the art in CI construction pertain to the selection of indicators, the identification of a proper system of weights for each dimension and each indicator and the adoption of an aggregation method that can account for such weights (Nardo et al. 2005; Cherchye et al. 2007; Davino and Romano 2013).

In this paper we exploit Multiple Factorial Analysis (Escofier and Pagés 1988) to synthesize all the observed indicators taking into account the role played by the different vulnerability dimensions. Since social vulnerability can be quite different according to the presence of heterogeneity (for example according to the different areas of a city) we exploit Quantile Regression (Koenker and Basset 1978) to measure if living in specific areas can play a different role in places characterised by low, medium or high vulnerability. The empirical analysis refers to the measurement of vulnerability in the Municipality of Rome according to a set of indicators constructed from the data of the fourteenth census of the Italian housing and population authority. The census data, collected at individual level, required an intensive pre-processing phase to obtain indicators referring to the neighbourhoods that represent the units of analysis of the paper.

All computations were done using the R package FactoMineR^1^ (Lê et al. 2008) dedicated to multivariate Exploratory Data Analysis and the XLSTAT software (XLSTAT 2017), a complete statistical add-in for Microsoft Excel.^2^

The paper is organised as follows. Section 2 defines the concept of social vulnerability while the methodology used to identify the dimensions of social vulnerability in urban spaces is described in Sect. 3. Section 4 introduces the main issues related to the CIs construction and the multivariate methods and models used in the empirical analysis. Data are presented in Sect. 5 from a univariate and bivariate point of view while Sect. 6 shows the results of the multivariate analysis. The paper concludes with some final remarks highlighting implications for policy in Sect. 7.

## Background: Social Vulnerability and the New Social Risks

Vulnerability can be described as ‘a life situation in which the autonomy and self-determination of individuals are permanently threatened by an unstable inclusion into the main system of social inclusion and distribution of resources’ (Ranci 2002). The crisis of these systems of integration and distribution, which are identified in the role played by the labour market, the family and the system of public welfare, gives rise to what Esping-Andersen (1999) identifies as a ‘new class of losers’: people living in households of people who are jobless, one-parent families, families on a single income, and people with disabilities. In this framework, it is interesting to observe individual, social and environmental factors that can determine poverty and a process of social isolation and impoverishment.

The concept of poverty, traditionally understood as a lack of sufficient income, now appears to be limited; the individual, her/his family dimension and information about housing and living zone become indispensable to identify the real conditions of social, economic and cultural inclusion (Negri and Saraceno 2004; Ranci 2002) and her/his capacity and opportunities to respond and recover from natural, environmental, socioeconomic and epidemic hazards.

The observation of new social risks (Ranci 2002; Taylor-Gooby 2004) has been enriched by the concept of social vulnerability (Ranci 2010), which has been substantially amended several times and adapted to different contexts. Several frameworks, model and assessment techniques have been provided following different approaches-. A first systematization is offered by Chambers (1989), who identifies two types of vulnerabilities: one external and one internal. The former refers to risks faced by individuals and families; the latter concerns the lack of means to counter these risks. Conversely, Moser (1998) identifies two dimensions in the context of the analysis of vulnerability: sensitivity, which is the force that the system is able to exercise so as to respond to an external event, and resilience, which is the ease and rapidity with which a system is able to react to negative effects caused by an external event. The second approach assigns a key role to the means that individuals possess. In particular, Moser argues that a greater amount of goods determines a lower vulnerability.

Watts and Bohle (1993) investigate vulnerability by employing three different dimensions of risk: exposure, capacity and potentiality. The first is the risk of being exposed to dangers; the second is the risk of not having sufficient and adequate capacity to face with the dangers; the third is the risk represented by the consequences of the events of crisis, shock and stress. This approach takes into account the dichotomy between internal and external vulnerability. The degree to which population is vulnerable to risks and hazards can be interpreted by considering the physical nature of hazards and the social characteristics of the population. There is a fundamental relationship among demographic, social and political characteristics of the population and place. For this reason several approaches explored how physical and social phenomena interplay with geographic characteristics: the exposure model investigates the conditions that determine vulnerability of people and place (Burton et al. 1993; Anderson 2000); a second model considers vulnerability as a social condition and measures the resilience of the population (Blaikie et al. 1994; Hewitt 1997; Cutter et al. 2008); a third approach integrates the previous two models (exposure and resilience) to analyse specific places and regions, and in particular the conditions that determine vulnerability of people and place (Kasperson et al. 1995; Cutter et al. 2003; Chakraborty et al. 2005; Cardona 2005; Tate et al. 2010; Dolan and Messen 2012), how specific individual and environmental characteristics determine the social amplification of risks (Kasperson et al. 1988), the population and policy response to risk (Cutter et al. 2003; Smit and Wandel 2006; Cardona 2005; Tate et al. 2010) and the disaster risk reduction (Kasperson et al. 1988; Cutter et al. 2003). Levernier et al. (2000) have emphasized how poverty rates can be different across geographic areas on the basis of person-specific and place-specific characteristics. Finally, Ranci (2002) identifies the difference between vulnerability and social exclusion in the degree of exposure of individuals to new social risks. He states that the concept of danger differs from that of vulnerability because the latter represents the level of exposure to the damage that can result from a negative event. However, events that can cause hazardous conditions are numerous and unpredictable, and for this reason, Ranci proposes a shift of perspective regarding most vulnerable people i.e. people that appear most vulnerable to the new social risks characterised by conditions of instability. The most vulnerable individuals are characterised by a persistent form of social and economic insecurity and with weak family and social networks. Thus, vulnerability can be seen as a persistent risk of marginalization, determined by a variety of economic, social, cultural and environmental factors: job insecurity, unemployment, low level of skill and education, chronic diseases and mental illness, disability, lack of residence permit, use of drugs and alcohol, housing deprivation, condition of overcrowding, lack of medical experts and medical services, ghettoization, domestic violence, etc.

For this reason, mapping social vulnerability can increase the understanding of population characteristics and their distribution at the urban scale, in order to identify areas with different capacity to react and respond to natural, socioeconomic and epidemic risks and to provide a useful tool for planning and managing the recovery and mitigation measures. It is important to note that, do date, there are no studies measuring and mapping social vulnerability at urban scale in Italy. Frigerio and De Amicis (2006) have analyzed the social vulnerability to natural hazards at national level focusing on Municipalities, while Didkovskyi et al. (2020) have concentrated their analysis on the specific topic of seismic hazard in Italian Municipalities.

## Measuring Social Vulnerability

The study of social vulnerability in the context of a plurality of human types can be addressed through the theoretical tools offered by the capability approach of Amartya Sen (1985, 1987). Investigating social vulnerability through this multidimensional approach means to interpret this condition as a form of incapacitation in converting capabilities into resources, tools and relationships. Sen defines capability as the capacity, ability and freedom to realise a functioning. Functionings represent what an individual can achieve and accomplish in his/her lifetime, and a person’s capability of living a good life is defined in terms of a set of valuable ‘beings and doings’ (Sen 1999). This approach enables an assessment of how individuals translate functionings into achievements. Capabilities delimit the space of freedom that an individual has in terms of choice - the reason why a limited capability set corresponds to a limited freedom of choice - which can determine a condition of deprivation. In this sense, vulnerability can be understood as a gradual loss of capabilities. Thus, the capability approach represents an innovative tool to describe and understand social vulnerability because it provides a theoretical basis for interpreting vulnerability as a condition in which individuals are affected by the lack of means and skills to convert resources into functionings.

To proceed with a practical implementation of the capability approach, we investigate seven dimensions of vulnerability, the result of a practical compromise in the data: housing, social context, education, labour market, mobility, social relations, economic well-being. Nevertheless, if the dimensions identified can be considered complete to evaluate the social condition of individuals and their level of social vulnerability (education, labour market and social and economic well-being), they will also be able to provide a detailed overview of the urban context in which these people reside (housing conditions, mobility, social context). To conclude the description of the social and urban context, mobility and access to the labour market represent fundamental sources of information. In addition, education level provides information on the degree of freedom that individuals are able to exercise in their own choices.

The literature on neighbourhood effects summarised by Galster (2012) can be considered as a theoretical basis supporting our choices because it highlights the relationship between dosage, in terms of community capability set, and response, understood as effective freedom to manage the current endowment set. The author identifies the origin of those effects in fifteen different mechanisms referred to endogenous and exogenous processes that characterises a place and his community, which are simplified into the following four groups:

1. Social-Interactive Mechanisms (Social Contagion, Collective Socialization, Social Networks, Social Cohesion and Control, Competition, Relative Deprivation, Parental Mediation)
2. Environmental Mechanisms (Exposure to Violence, Physical Surroundings, Toxic Exposure)
3. Geographical Mechanisms (Spatial Mismatch, Public Services)
4. Institutional Mechanisms (Stigmatization, Local Institutional Resources, Local Market Actors)

The dimensions in the first group were selected on the basis of numerous studies identifying a strong predictor of a variety of deviant behaviours in peer effects and negative social models in disadvantaged neighbourhoods, a mechanism that Galster (2012) describes as a real kind of social contagion that is akin to an ‘epidemic’.

Table 1 links the seven previously described dimensions of the vulnerability to the mechanisms proposed by Galster (2012). An analysis of individual capabilities (being educated, having a good job and being safe) needs to be integrated with an investigation of the social and environmental context and by the individual’s social network. In order to highlight the mechanisms of social interaction, the characteristics of the social context (B) and the availability of social networks (F) must be considered. The second group has been summarised through the identification of the characteristics of space, namely the characteristics and living conditions of households (A). The third group identifies the factors that could negatively affect people’s quality of life in the context of the labour market (D) and mobility services (E). Finally, while the fourth group relates to the institutional mechanism, the housing and population census does not allow the identification of useful data in rebuilding the role played by institutional resources.

**Table 1:** The mechanisms of neighbourhood effects proposed by Galster (2012) are linked to the dimensions of social vulnerability

Mechanism	Dimension
Social-interactive mechanisms	(B) Social context
	(C) Education
	(F) Social relation
	(G) Economic well-being
Environmental mechanisms	(A) Housing
Geographical mechanisms	(D) Labour market
	(E) Mobility
Institutional mechanisms	–

## Methods

### Composite Indicators Construction

The proposed empirical analysis aims to measure the degree of vulnerability in the districts of the city of Rome taking into account the seven dimensions of vulnerability. Since the object of analysis cannot be directly observed and measured, it is necessary to identify and summarise individual indicators (quantitative/qualitative measures observed on a set of units) into a single index, namely a composite indicator (CI). Increasingly, political decisions aimed at sharing financial resources are based on the use of CIs as reference tools.

Notwithstanding their potentiality, CIs remain controversial as they are often lacking in standard and objective construction methodology. Whatever the application context, the construction of CIs involves stages in which several decisions have to be taken (Nardo et al. 2005; Davino and Romano 2013). These decisions have a strong influence on the quality of CIs. Moreover, CIs may be misused if the construction process is not transparent. The first requirement pertains to the characterisation of the dimensions underlying the concept to be measured (Land et al. 2012). Once these have been identified, indicators measuring each dimension must be specified. The selection of indicators and weights should be done very carefully as a CI could exacerbate disagreements rather than focus minds. A pre-processing of the univariate indicators is then performed to deal, for example, with missing values and with the coding and scale transformation of raw values (Thiessen 1997). Finally, several aggregation methods and systems of weights (Hagerty and Land 2007) can be adopted.

### Multivariate Methods and Models

The empirical analysis proposed in this paper exploits the potentialities of two multidimensional data analysis methods: principal component analysis (PCA) and multiple factorial analysis (MFA) (Lebart et al. 1984; Escofier and Pagés 1994).

The paper follows the French school of “analyse des données” (data analysis) led by Jean-Paul Benzécri (1982) who encouraged the idea of “letting the data speak for themselves” rather than Exploratory/Confirmatory Factorial Analysis (Thurstone 1931; Jolliffe 2002).

PCA dates back to the pioneering work of Pearson (1901), which focused on the simultaneous analysis of several quantitative variables. PCA aims to identify a reduced subset of latent variables, which are not observed but obtained as a weighted combination of the original variables. The procedure exploits the correlation between the observed variables, enables a reduction in the complexity of the multidimensional data and eliminates redundant information.

PCA can provide a valid support in most of the phases of CI construction. First, as it is based on the correlation between variables, it can help with the selection of indicators. For example, in case of high collinearity, the results do not change if one of the redundant variables is eliminated. Moreover, in the pre-processing phase, PCA can always be carried out using standardised variables, thus avoiding the problem of handling variables expressed in different units of measurement. Second, the identified latent variables (commonly named principal components or factors) are uncorrelated, each one explaining a decreasing quota of information. The first principal component can be acquired as the CI’s object of study. Noteworthy, it should explain a sufficient percentage of variability (measured by the first eigenvalue). The ability to explore the remaining principal components is a further strength of the method: it is possible to visualise in two dimensions the relationships among variables and units, with each dimension being one of the extracted latent variables. Finally, each PCA is a weighted linear combination of the original indicators whereby the system of weights, named the eigenvectors, expresses the role and importance of each indicator in designing the synthetic index.

MFA (Escofier and Pagés 1988, 1994) can be used to analyse observations described by several ‘blocks’ or sets of variables. MFA seeks the common structures present in all or some of these sets and provides results that can be used to study the relationship among observations, variables and blocks at the same time.

MFA is performed in two steps. First, a PCA is performed on each block of variables, which is then ‘normalised’ by dividing all its elements by the square root of the first eigenvalue obtained from the PCA. This type of weighting ensures that blocks that include more variables do not weigh too much in the analysis. Second, the normalised blocks of variables are merged to form a unique matrix, and a global PCA is performed on this matrix. The individual data sets are then projected onto the global analysis to analyse commonalities and discrepancies.

In the CI framework, MFA provides both a synthesis of each dimension and the final CI. The former is obtained as a weighted linear combination of the original indicators while the latter is the first factor of the PCA performed on the weighted data matrix. It results that MFA is a particular weighted PCA where the block structure is preserved because all the variables in the same block have the same weight. No group can generate the first axis of the global analysis on its own and the first dimension is the ‘consensus variable’, the most linked to all the variables. From the point of view of policy-makers, this means that it is possible to identify the role played by each indicator and by each dimension on the final CI.

The distribution of the global CI identified by the MFA can be analysed to identify possible differences in the urban space. The paper proposes the use of Quantile Regression (QR) to analyse if the impact of living in a given geographic area is different for the different levels of the vulnerability. Quantile regression, as introduced by Koenker and Basset (1978), may be considered an extension of median regression and a complementary method to ordinary least squares regression because QR is based on the estimation of a set of conditional quantiles of a response variable as function of a set of covariates (Davino et al. 2014). QR is emerging more and more as a methodology complementary to ordinary least squares regression in several applicative contexts, from ecology to economics, marketing and social sciences. Although different functional forms can be used, the paper will refer to linear regression models. It is worth to recall that, no parametric distribution assumptions are required for the error distribution. The parameter estimates in QR linear models have the same interpretation as those of any other linear model. The intercept measures the dependent variable value deriving from setting to zero all the regressors at the corresponding conditional quantile. Each slope coefficient can be interpreted as the rate of change of the given conditional quantile of the dependent variable distribution per unit change in the value of each regressor, taking the other regressors constant. For each quantile, a regression model is estimated. As a consequence, the estimated values of the response variable conditioned to given values of the regressors, reconstruct the conditioned quantiles of the dependent variable.

## Data

The empirical analysis is based on data from the fourteenth census of the Italian housing and population authority (2001) and compiled by the Italian Statistical Institute (ISTAT)^3^. In the Municipality of Rome more than one million families and more than 2.5 million individuals were counted in the fourteenth census. The 13,240 units of census sections have been aggregated in 115 toponomy areas that represent the four groups of neighbourhoods of the Municipality of Rome^4^.

On the one hand, since the aim of this paper is to measure social vulnerability as an interrelation of geographic location, the units of analysis are represented by the 115 neighbourhoods of the Municipality of Rome. On the other hand, as the family and the individual are the units of analysis in the census data, it was necessary to construct a set of indicators referring to the neighbourhoods. Thus, a pre-processing of the census data was carried out in order to summarise the indicators observed for the individuals and families belonging to each neighbourhood. The list in the Appendix describes how each indicator was obtained and which data from the census were used. This phase was particularly laborious because the transfer from analytic to aggregated data was not anticipated, and there was no unique solution. For example, the job declared by each unit can be aggregated with respect to each neighbourhood by deriving the percentage of persons with a given job. In the case of the variables referred to as home features (presence of a kitchen, heating appliances, shower, etc.), the aggregation of the home features of each individual can be performed in several ways: the average number of features per home in the neighbourhood, the percentage of each feature in the neighbourhood, etc.

The final result of the pre-processing phase was a data matrix in which each row represents a neighbourhood (112 in total)^5^ and each column an indicator (21 in total).

**Fig. 1:**
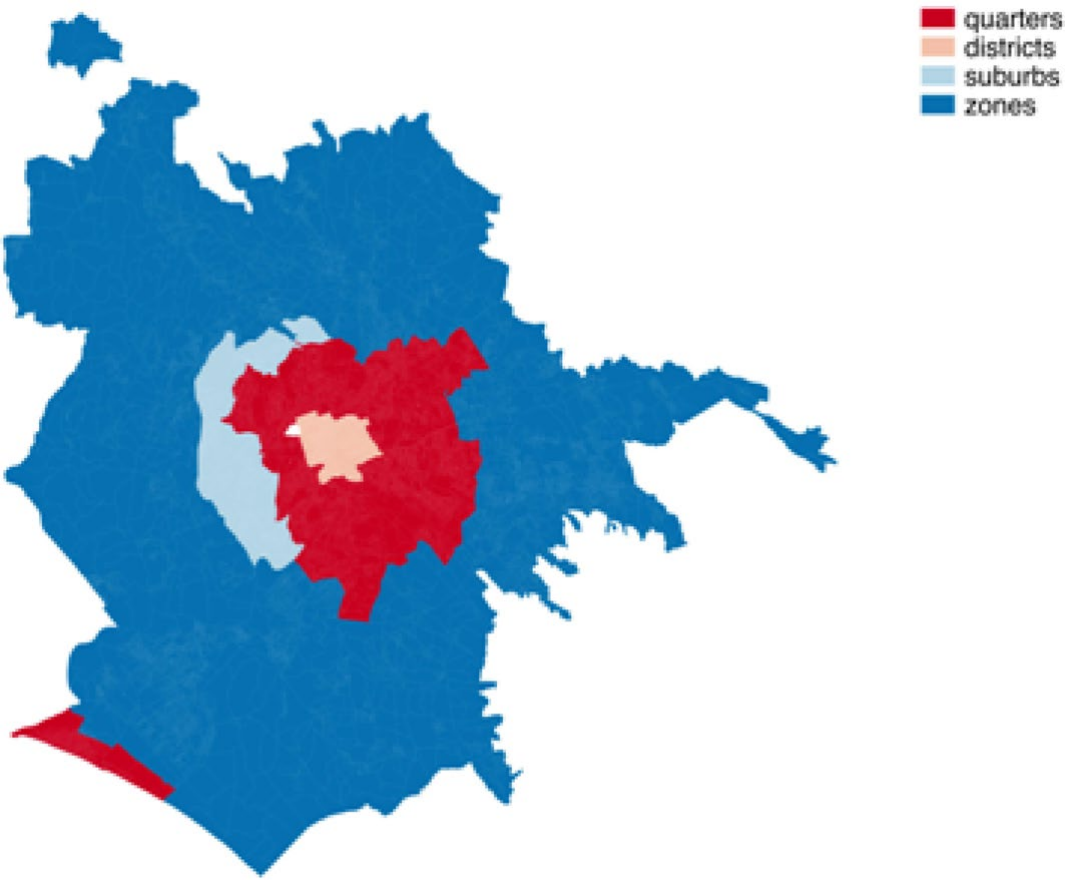
Classification map of neighbourhoods of the Municipality of Rome in four areas, starting from the historic centre (districts and quarters) and moving towards the outer part of the city (suburbs and then zones)

In order to measure social vulnerability in the Municipality of Rome, it is important to take into account that the 112 neighbourhoods are classified into four areas: quarters (located in the historic centre of the city, mostly within the Aurelian Walls, except for Prati and Borgo) districts (surrounding the historic centre of Rome outside the Aurelian Walls), suburbs (located in the suburban area of the city) and zones (Agro Romano, the outer parts of Rome’s municipality). Table 2 shows the number and percentage of neighbourhoods in the four areas (the labels in the second column are used hereinafter in graphs and tables) while Fig. 1 depicts the map of the city, highlighting the four areas. From the map, it is evident that the classification reproduces the structure of the municipality, moving from the centre (districts) to the fringes of the urban area (zones).

**Table 2:** Distribution of the 112 neighbourhoods according to the four areas in which the municipality of Rome is classified

Area	Label	Frequency	%
District	R	22	19.6
Quarter	Q	35	31.3
Suburb	S	6	5.4
Zone	Z	49	43.8
Total		112	100.0

The data matrix was partitioned in columns because the set of the indicators was grouped into the seven dimensions identified from the previously described literature on the topic (see Sect. 3):

A. Housing
B. Social context
C. Education
D. Labour market
E. Mobility
F. Social relations
G. Economic condition

The capital letter before the name of the dimension will be used in tables and graphs as labels. All the indicators but A1 are expressed in percentages.

**Table 3:** Key descriptive univariate statistics for the twenty-one indicators related to seven vulnerability dimensions (capital letters in the first column are the labels previously assigned to the dimensions): minimum (Min), first quartile (Q1), Median (Median), average (Mean), third quartile (Q3), maximum (Max), standard deviation (SD), variation coefficient (CV), asymmetrix index (Asym)

	Min	Q1	Median	Mean	Q3	Max	SD	CV	Asym
A1	5.2	5.7	5.8	5.7	5.8	5.9	0.1	1.8	$-2$
A2	1.5	5.3	7.8	8.8	10.5	23.8	4.5	50.9	1.1
A3	29.2	37.1	43.1	44.6	50.3	75.1	9	20.3	0.7
B1	1.3	3.3	4.2	4.4	5.5	7.1	1.3	30.5	0
B2	37.2	48	51.7	50.9	54.1	62	4.8	9.5	$-0.5$
B3	30.5	36.1	38.6	39.1	42	50.3	4.3	11	0.5
B4	2.5	4.1	5.2	5.6	6.5	18.4	2.4	43.4	2.6
C1	20.2	29.2	40.2	39.2	48.2	58.5	10.7	27.4	$-0.1$
C2	20.5	29.7	33.3	32.7	35.7	43.5	4.7	14.2	$-0.5$
C3	0.5	1.1	1.8	2	2.9	4.7	1.1	51.8	0.4
C4	0.2	0.5	1	1.1	1.6	3.4	0.8	66.8	0.8
D1	33.2	37.8	39.7	39.9	41.6	49.1	3.1	7.7	0.3
D2	43.5	60.1	65.8	67.3	76.4	85.8	10.3	15.3	0
D3	3.1	5.1	6.6	6.7	8.1	13.1	2	29.8	0.6
E1	10.1	18.3	22.9	22.6	25.9	32.7	4.9	21.7	$-0.2$
E2	36.9	55.2	63.2	61.7	68.2	83.9	10	16.2	$-0.3$
E3	2.8	8.7	14.4	15.7	19.6	42	9.1	57.9	1
F1	5.1	7.4	10.4	12.1	17	27.1	5.6	46.3	0.7
F2	45	51.2	52.7	53.8	56.8	63	3.8	7.1	0.5
G1	38.1	56.6	64.6	63	72.2	87.2	11.5	18.2	$-0.4$
G2	0	4.1	8.1	12.1	17.1	52.8	11.5	94.5	1.3

The main descriptive statistics (Table 3) show differences in the central tendency, in the variability and in the shape of the indicator distribution. Looking at the variation coefficients (column CV in Table 3), it results a moderate variability for most of the indicators, with the exception of G2, F1, E3, C4, C3, B4 and A2. In such cases, the features of the neighbourhoods are not homogeneous. In particular, the variation coefficient is very useful for comparing variability of the distributions that differ considerably in terms of averages and therefore it allows us to overlook the territorial specificities of certain phenomena. It is also interesting to highlight that G2, B4 and A2 are characterised by positive asymmetry (column Asym in Table 3): most neighbourhoods have low values for these indicators; there are few neighbourhoods with very high values while A1 shows a negative asymmetry. This indicates that a reduced number of neighbourhoods shows a high critical level in relation to some indicators. In particular, we can observe the concentration of the social housing sector, such as the presence of overcrowded dwellings and high levels of family vulnerability in a limited number of neighbourhoods. This evidence may suggest the existence of a spatial and social differentiation in the city, which is determined by the characteristics of people living there and by their economic and living conditions.

**Table 4:** Averages for the indicators according to the area

	Quarter	District	Suburb	Zone	Mean
A1	5.8	5.68	5.74	5.7	5.7
A2	9	6.76	12.24	9.04	8.8
A3	40.7	35.67	44.77	51.33	44.6
B1	5.12	5.64	4.45	3.31	4.4
B2	52.78	53.2	50.68	48.48	50.9
B3	37.77	35.68	39.38	41.54	39.1
B4	4.32	5.48	5.46	6.66	5.6
C1	37.48	28.27	41.26	45.02	39.2
C2	34.1	34.15	31.86	31.13	32.7
C3	2.23	3.28	1.91	1.35	2.0
C4	0.84	0.48	1.33	1.62	1.1
D1	38.82	42.76	39.09	39.48	39.9
D2	68.71	81.63	67.44	59.84	67.3
D3	6.13	5.19	7.86	7.74	6.7
E1	23.94	21.45	18.33	22.7	22.6
E2	59.17	49.12	71.48	67.95	61.7
E3	16.9	29.45	10.18	9.37	15.7
F1	13.53	20.87	10.02	7.44	12.1
F2	54.11	59.64	53.15	51.03	53.8
G1	65.75	51.2	63.89	66.21	63.0
G2	13.49	11.01	15.18	11.26	12.1

A univariate analysis of each indicator through the area means and the global mean of the whole municipality (Table 4) shows some differences in the living conditions within the four areas. Quarters and districts are characterised by better conditions; however, the dimensions ‘Mobility’, ‘Social relations’ and ‘Economic condition’ register average values higher than the global mean for the following indicators: E1 public mobility, E3 walking, F1 no cohabitants, F2 no partner, G1 ownership and G2 social housing. These results are not easy to explain at this stage of the analysis; the global view provided by the MFA will help the interpretation.

## Results

### Explorative Data Analysis

MFA is based on several PCAs each one carried out on the set of indicators relating to each dimension. It is thus advisable that the multivariate analysis is preceded by a check on the unidimensionality of each dimension. Such a requirement is crucial because MFA takes into account the first component extracted by each PCA.

There are three main tools commonly used to check for unidimensionality: the principal component analysis of each dimension, Cronbach’s $\alpha$ and the Dillon–Goldstein’s $\rho$. A dimension is essentially unidimensional if the first eigenvalue ($\lambda _1$) is larger than 1 and the second one ($\lambda _2$) smaller than 1, or at least very far from $\lambda _1$. A dimension is also considered unidimensional when Cronbach’s $\alpha$ and/or Dillon–Goldstein’s $\rho$ are larger than 0.7. The statistics for checking the unidimensionality of each dimension are presented in Table 5 (from the third to the sixth column). With the exception of Housing, where just the Cronbach $\alpha$ is not satisfactory, and of Mobility, where the second eigenvalue is slightly higher than 1, all the statistics lead to an acceptance of the unidimensionality of all the dimensions. Moreover, Dillon–Goldstein’s $\rho$ is considered by some authors (e.g. Chin 1998) to be a better indicator of the unidimensionality of a dimension than Cronbach’s $\alpha$.

**Table 5:** Main statistics related to each dimension: name (Label), number of indicators (#vars), indexes to check for dimension unidimensionality (Cronbach’s $\alpha$, Dillon–Goldstein’s $\rho$, first eigenvalue and second eigenvalue), percentage of variability explained by the first two factors of the PCA on each dimension (F1 and F2)

Label	#vars	$\alpha$	$\rho$	$\lambda _1$	$\lambda _2$	F1	F2
Housing	3	0.57	0.78	1.62	0.78	53.9	25.9
Social context	4	0.86	0.91	2.85	0.65	71.2	16.3
Education	4	0.94	0.96	3.43	0.4	85.7	10.1
Labour market	3	0.77	0.87	2.07	0.59	69.0	19.6
Mobility	3	0.67	0.83	1.94	1.06	64.7	35.3
Social relations	2	0.95	0.97	1.9	0.1	94.9	5.1
Economic condition	2	0.75	0.89	1.6	0.4	79.8	20.2
MFA						43.4	17.7

Results from PCA on each of the seven dimensions are satisfactory with respect to the percentage of variability explained by the first factor (column ‘F1’ in Table 5). This allows us to consider each first factor derived from the seven PCAs as a reliable CI of the related dimension. Table 5 also shows the percentage of variability explained by the second factor (column ‘F2’), which is useful in case of a two-dimensional representation of the results. The MFA is able to explain more than 60% of the variability for the two first factors.

A crucial step in the interpretation of the results is to exploit the graphical potentialities of the factorial methods and, in particular, of MFA, which enables a visualisation of the relationships among the dimensions as well as among the indicators and the statistical units.

**Fig. 2:**
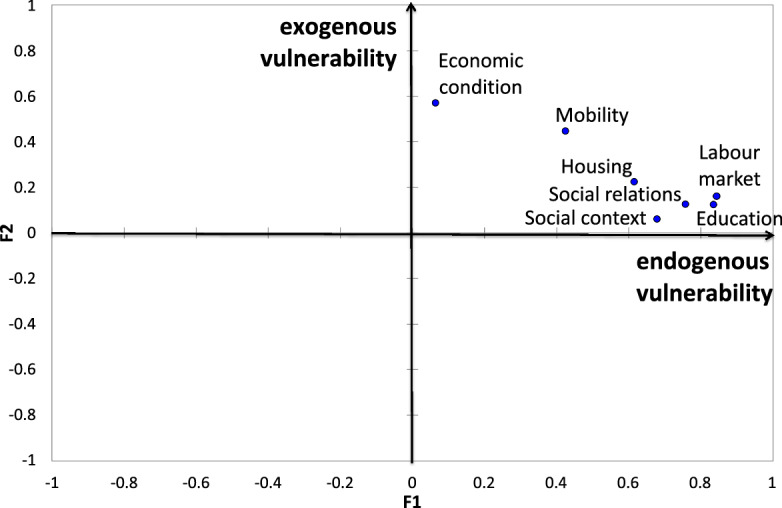
Representation of the seven dimensions of the vulnerability on the MFA first factorial plane. The coordinates of each group are obtained according to the correlations between the variables belonging to the group and the first principal component obtained in the global analysis

The dimensional representation in a two-dimension space (Fig. 2) shows that five of the seven considered dimensions are highly correlated with the first factor while ‘economic condition’ and ‘mobility’ are mainly correlated to the second factor. This result can be interpreted by taking into account that the latter two dimensions have a strong relationship with the space. For example, the possibility of becoming an owner relies on the market price, which is in turn related to the zone in which the house is located. Another example could be the use of private vehicles to reach the workplace, which could be related to the availability of public transport. The representation of the seven dimensions of vulnerability on the MFA’s first factorial plane suggests the presence of two different CIs linked to the vulnerability: the first representing endogenous vulnerability, mainly related to the individual, domestic and housing features, and the second representing exogenous vulnerability, mainly related to the spatial location.

**Fig. 3:**
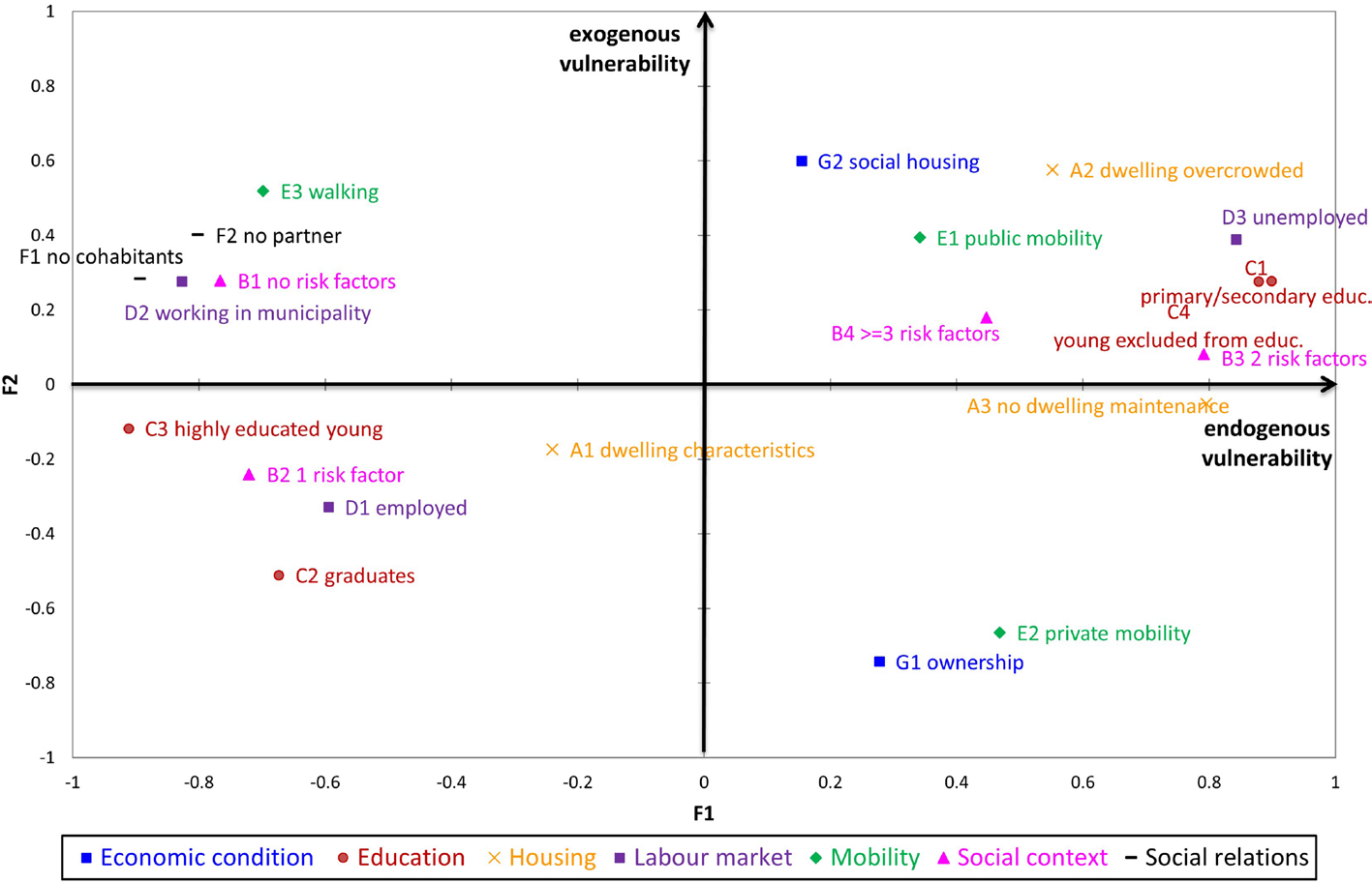
Representation of the indicators on the MFA first factorial plane. The plot is obtained through a weighted PCA performed on the whole set of indicators but preserving the group structure. All the indicators in the same group have the same weight equal to the reciprocal of the first eigenvalue obtained from the PCA performed on the group it belongs to. The shape and the color of the points allow to classify the indicators according to the dimension. (Color figure online)

Figure 3 shows the 21 indicators projected on the first MFA factorial plane. Endogenous vulnerability increases with the increase in deprivation of residents’ capabilities (low level of education and unemployment), social context (high family vulnerability), physical deterioration of buildings (no dwelling maintenance) and housing conditions (overcrowded dwelling). Conversely, exogenous vulnerability is linked to the characteristics of the neighbourhoods. In particular, it is the result of a spatial differentiation. As noted earlier, the concentration of marginal groups can lead to changes in the quality of the neighbourhoods, the stigmatisation of the area and the exclusion of people living there. For this reason, we considered the concentration of social housing as an element of exogenous vulnerability because we assumed that the households in these areas are economically and socially homogeneous and that segregation and socio-spatial inequality are mutually self-perpetuating processes.

**Fig. 4:**
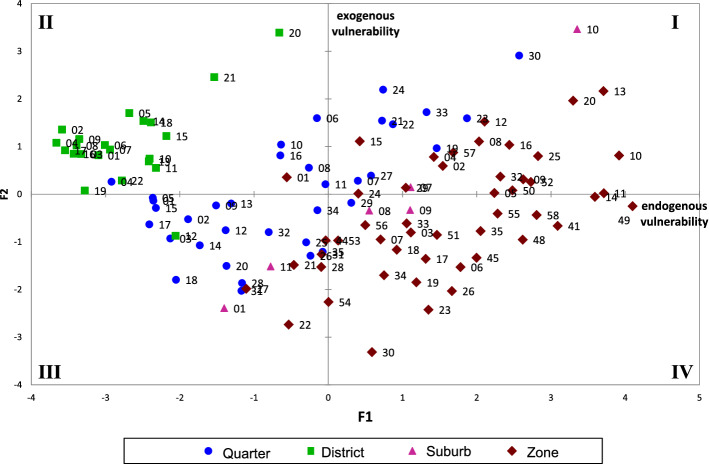
Representation of the neighbourhood on the MFA first factorial plane. The shape and the color of the points allow to distinguish neighbourhoods according to the area defined in Table 2 and Fig. 1. (Color figure online)

The neighbourhood representation (Fig. 4) must be interpreted by considering that endogenous vulnerability becomes higher and moves from left to right and that exogenous vulnerability moves from the bottom to the top of the graph. With respect to the classification of the neighbourhoods in the four areas, this results in an increase in the endogenous vulnerability in moving from districts to quarters, suburbs and zones while more variability across the areas holds in cases of exogenous vulnerability.

In order to identify the most critical neighbourhoods, the plot is divided into four quadrants numbered starting from the top right and counter-clockwise, as in current practice. The first quadrant is characterised by high vulnerability, both endogenous and exogenous, and therefore is a critical region. Also the second and fourth quadrants should be monitored. In particular, we can observe that the highest level of vulnerability is recorded in the eastern and western suburbs of Rome and more generally in correspondence with the most peripheral areas of the city.

**Fig. 5:**
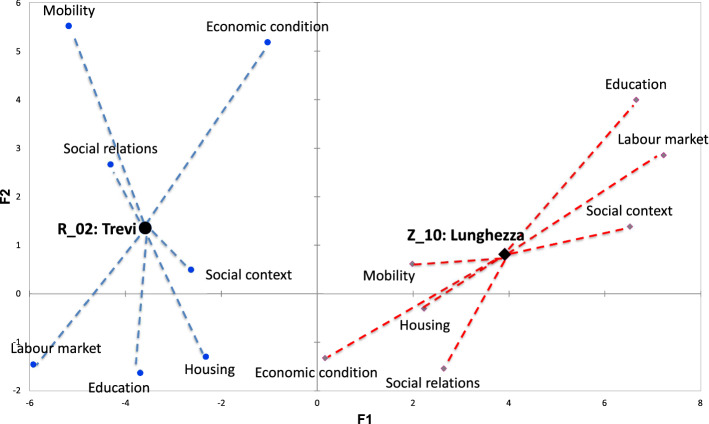
The representation of two sample neighbourhoods on the MFA first factorial plane. Lunghezza is located in a zone (fringe of the urban area) and Trevi in a district (center of the city)

MFA also allows to explore how the different dimensions influence the position of each statistical unit on the map in Fig. 4. Figure 5 shows two sample neighbourhood Lunghezza and Trevi. The former is located in a zone (fringe of the urban area) and the latter in a district (center of the city). The representation is named graph of partial individuals because the position of each neighbourhood is linked through dotted lines to its position on the factorial planes obtained considering just the indicators of a dimension at a time. It results that each neighbourhood is the barycenter of its partial points. The partial representation shows that Lunghezza is mainly characterised by a high vulnerability with respect to the aspects related to education, labour market and social context, while Trevi is mainly influenced by factors related to its spatial position (Economic condition and Mobility).

### Linking Vulnerability to Urban Space

**Fig. 6:**
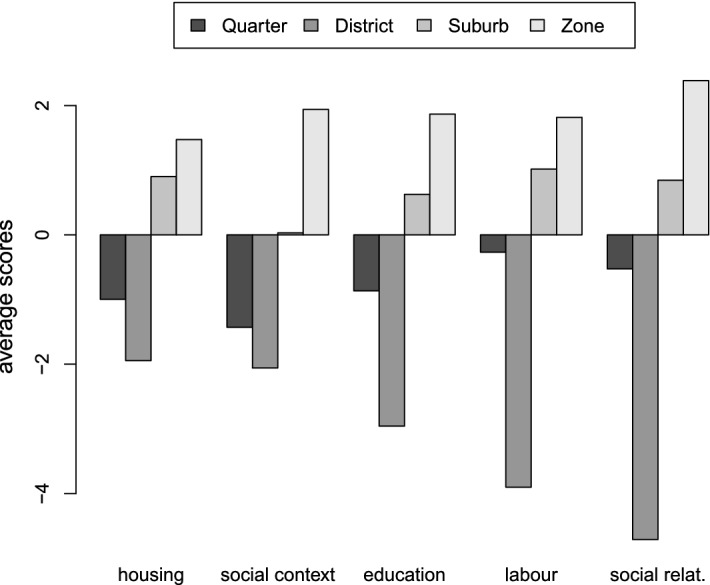
Endogenous vulnerability (first factor of the MFA) average values according to the area. The interpretation of the barplots must consider that the index is a standardised variable and thus with an average equal to zero

**Fig. 7:**
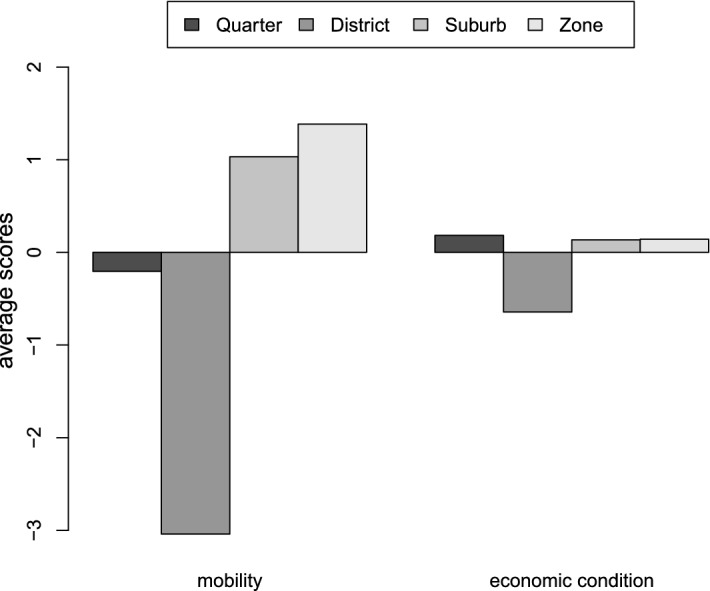
Exogenous vulnerability (second factor of the MFA) average values according to the area. The interpretation of the barplot must take into account that the index is a standardised variables and thus with an average equal to zero

A post-analysis of the results provided by the MFA can be carried out to explore possible differences in the territorial areas. A first in-depth study can take into account the average values of the endogenous and exogenous vulnerability in the four areas (Figs. 6 and 7). It is worth noting that the two CIs, endogenous and exogenous vulnerability, are standardised variables and thus with an average equal to zero. There is a considerably generalised behaviour, below the general average, in the quarters and districts. Such a feature is particularly evident in districts with respect to labour, social relationships and mobility.

Endogenous vulnerability increases more than exogenous vulnerability by moving from districts to zones. These trends can be explained by combining the MFA results (Fig. 3) to the mean values of the indicators (Table 4). On the one hand, the upper part of the second factor is mainly characterised by neighbourhoods showing high values of G2 social housing and lower values of G1 ownership. On the other hand, the average values of the previously cited indicators for the neighbourhoods in the districts are lower than the total average. This means that there is some underlying feature, not included in the data set, characterising the neighbourhoods in the districts. The percentage of rented houses could be a possible explanation because it is well known that these areas are quite expensive; it is also reasonable to suppose that most of the population lives in houses for rent. This explanation is supported by positive conditions in the area with respect to education and the labour market (in Table 4, average values higher than the total average).

**Fig. 8:**
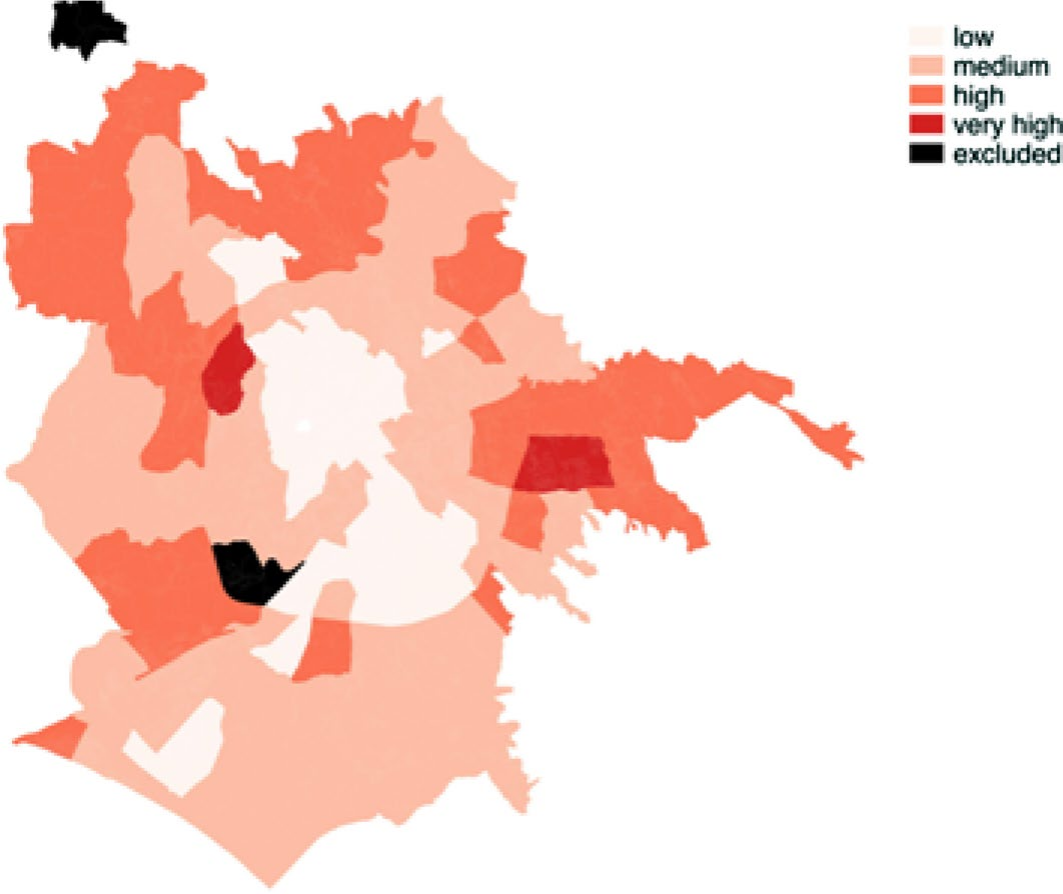
Map of the Municipality of Rome according to the vulnerability index obtained as a weighted combination of the first and second factor of the MFA measuring, respectively, the endogenous and exogenous vulnerability

A final ranking of the neighbourhood according to vulnerability is also provided (Fig. 8)^6^. This final CI was obtained as a weighted combination of endogenous and exogenous vulnerability, measured respectively through the first and second factor of the MFA. The weights are represented by the percentage of variability explained by each factor. The map shows greater vulnerability in the east, west edge and partially in the north part of the city. Vulnerability decreases when moving from the edges to the city centre, thus confirming worse living conditions in marginal areas and a socio-spatial hierarchy in the city of Rome.

**Fig. 9:**
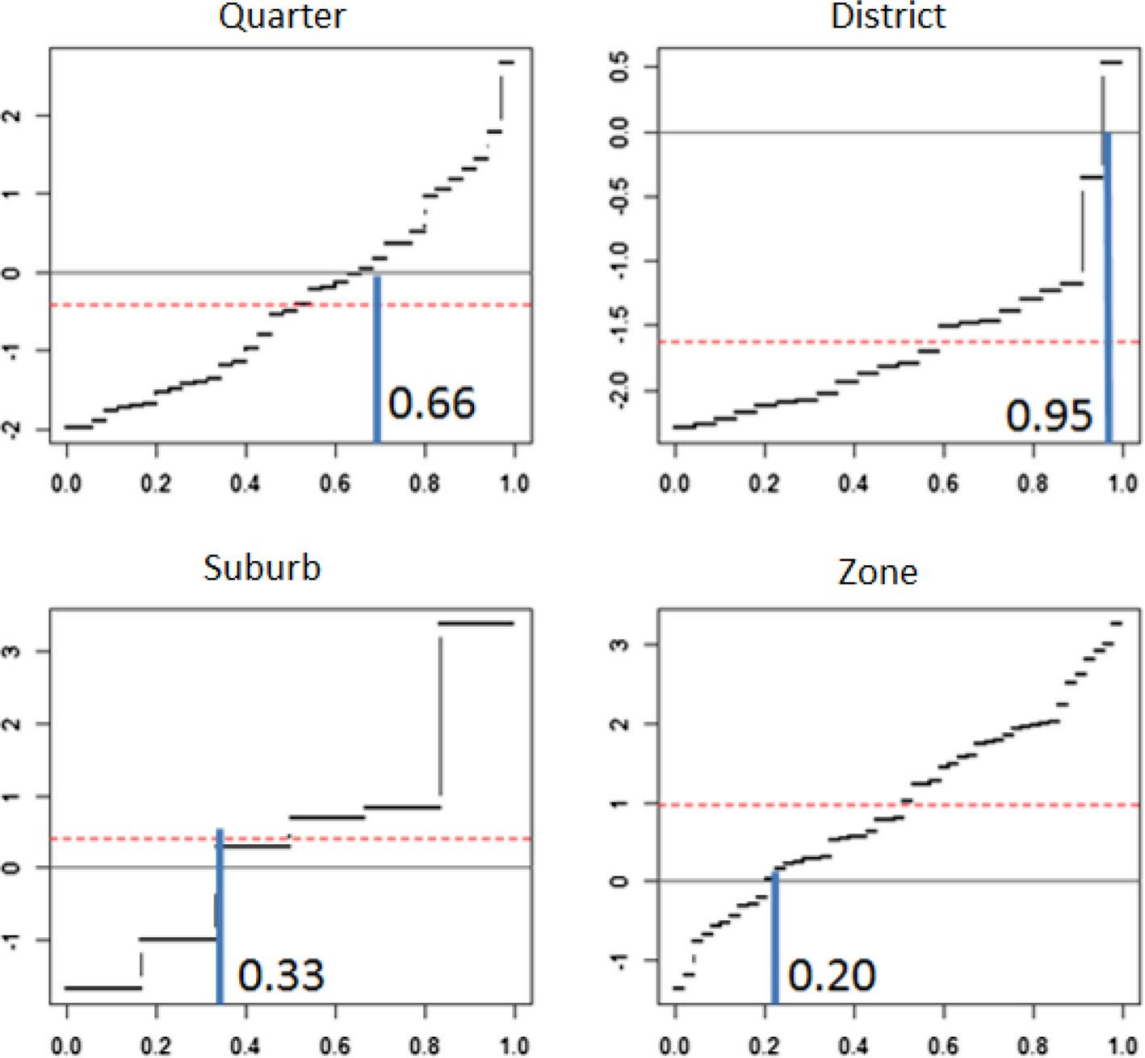
QR coefficients measuring the impact of the different areas on the global vulnerability. Coefficients are obtained through a Quantile Regression aiming to explore the effect of the areas on the different levels (quantiles) of the vulnerability

Exploring differences among the statistical units on the basis of the average values can be limiting hiding a possible heterogeneity in the urban space. Considering the vulnerability CI, it results that, as expected, the averages values in districts and quarters are below the global average (equal to zero) and above in case of suburbs and zones (vulnerability averages: $\hbox {Q}=-0.41$, $\hbox {R}=-1.63$, $\hbox {S}=0.41$, $\hbox {Z}=0.97$). If the whole distribution of the vulnerability is explored, it is possible to discover significant differences among the areas. At this regard the exploration of the quantiles of the vulnerability conditioned to each area are analysed. Figure 9 shows the trends of the coefficients of several quantile regressions estimated considering the total vulnerability as response variable and the area transformed into four dummy variables as regressors. This is a very simple model which, in essence, provides a comparison among the conditional quantiles but allows to analyse the impact of the area on the different levels of the vulnerability. The horizontal axis displays the quantiles and the vertical axis the coefficients estimated at each quantile. The dotted lines represent the conditional means, which discriminates the areas into two groups: below the average in case of areas quarters and districts and above for suburbs and zones. Considering the quarters, it is interesting to note that living in that area allows to reduce the vulnerability but this is true up to the 66th percentile of the distribution of the vulnerability. The vertical solid lines highlight the thresholds between negative and positive effects played by living in an area characterised from vulnerability. It is interesting to note that these thresholds are very different in the four areas, moving from 0.20 in case of the zones to 0.95 in case of the districts.

## Concluding Remarks and Further Developments

This paper aimed to highlight the different dimensions of social vulnerability and their impact on the formation of the urban structure and morphology of the city of Rome. The social characteristics of the urban space revealed the existence of a spatial fracture within the city. We can observe areas characterised by a strong presence of vulnerable individuals and areas in which vulnerability is very limited. The concept of vulnerability has been used in this research to map those individuals who demonstrate the superposition of different risk factors, although not necessarily poor or excluded. In particular, the research has highlighted a non-homogeneous spatial structure of the city of Rome: we recorded different levels of vulnerability in relation with the seven dimensions investigated, all of which made a different contribution to the overall condition of vulnerability. As we have seen, the vulnerability map provides information about the geographical distribution of the phenomenon. More specifically, the peripheral areas are the most affected by the vulnerability condition, and we can observe a high spatial segmentation characterised by a bi-partition in the city - centre and periphery: low vulnerability at the centre and high vulnerability in the eastern and western peripheral areas.

Another interesting finding of the research is the presence of two different types of vulnerability: one characterised by factors of an endogenous nature (individual, domestic and housing features) and the second by factors of an exogenous nature (mainly related to those dimensions that mostly depend on the spatial location).

The study of vulnerability developed in the paper has required an investigation of both the individual, with his capabilities, and the space. The former takes into account topics such as level of education, access to the labour market and housing, as well as the family structure and social context. The latter is seen as a crucial conversion factor of individual capabilities in terms of achievement. In this perspective, the results of the study reveal the existence of a socio-spatial segregation phenomenon in some districts of the city of Rome: the presence of spatial inequalities in individual opportunities to convert capabilities and resources into achievements is highlighted by the concentration of the most vulnerable individuals in suburban areas. The results show that the concentration of vulnerable individuals determines the characteristics and conformation of the space and their hierarchisation. The very fact of residing in these areas represents an additional risk for individuals because it determines a limitation of freedom whereby individuals can access the means and resources required to convert their skills into realised functionings and a potential reduction’s capacity to cope with the risks.

The key strengths of this research are represented by the observation of the spatial distribution of the dimensions of vulnerability and by the identification of the more vulnerable geographical areas. Moreover, the analysis was conducted using census data; thus, the results can be considered a faithful picture of vulnerability in the city of Rome. Another element of interest is represented by the opportunity to rank the neighbourhoods according to a vulnerability index and to discriminate the city areas between endogenous and exogenous vulnerability.

The different level of vulnerability registered within the city of Rome, in relation to each dimension analysed, allows to highlight why the risks can affect in a different way and intensity the residents. In addition, mapping exogenous and endogenous vulnerability (Galster 2012) provides a risk assessment and management tool and allow to planning prevention intervention and mitigation taking into account the specific characteristics of the territory and the population.

The research also has some limitations: the choice of a synthetic index determines a loss of a part of information. In addition, the absence of information relating to certain dimensions of vulnerability (such as income, institutional mechanisms and spatial features) determines that they remain unexplored. Finally, the limited availability of geocoded data limits the analysis of vulnerability at urban scale.

The policy implications arising from tackling these specific issues at the urban level are the necessity to promote access to individual conversion factors such as education and training; the reduction of social inequalities in accessing high-quality public services to improve the ability to cope with.

Further developments concern the improvement of the census data with administrative information that can be useful to expand the dimensions of vulnerability and the monitoring of the results over time using the most recent census data. This means that from a practical point of view, the research results could be used by policy-makers to detect the state of vulnerability in the different zones of the city and to identify the proper leverages to improve life conditions in each zone.

## Appendix

List of individual (ID), family data and neighbourhood indicators:

- A1 dwelling characteristics: average number of equipment within each unit of analysis
  (ID—presence or absence: dining room with kitchen, toilet, bath or shower, sanitary hot water, drinking water, heating system)
- A2 dwelling overcrowded: percentage of individuals who occupies less than 15 square meters, based on the total population of each unit of analysis
  (ID—presence or absence of living comfort ($\ge 15\,\hbox {mq}$))
- A3 no dwelling maintenance: share of dwellings that, within each unit of analysis, has not benefited from maintenance during the last 10 years
  (ID—Presence or absence of maintenance during the last 10 years for structural and nonstructural elements of the dwelling)
- B1 no risk factors, B2 1 risk factor, B3 2 risk factors, B4 $\ge 3$ risk factors: percentage of individuals who, within the same unit of analysis, does not show any of the following familial risk factors: belonging to a large family (5 members), to a single parent families; to a family with a single breadwinners or it is a single elderly person; in legal separation or divorce; a foreigner
  (ID—Household size; household type; age; professional status; marital status; citizenship)
- C1 primary/secondary education: Level of primary and secondary education
  (ID—Education level currently attended)
- C2 graduates: percentage of individuals who have achieved a level of education equivalent to upper secondary qualification
  (ID—Education level currently attended)
- C3 highly educated young: percentage of individuals between 30 and 34 years who, within each unit of analysis, has obtained a university degree qualification
  (ID—Education level currently attended; age)
- C4 young excluded from education: percentage of individuals between 18 and 24 years old with only a lower secondary education who, within each unit of analysis, are not in further education and training
  (ID—Attending a training course; education level currently attended; age)
- D1 employed: percentage of individuals employed from the total population within the same unit of analysis
  (ID—Professional status)
- D2 working in municipality: percentage of individuals who, within the same unit of analysis, working within the same municipality
  (ID—Habitual place of work)
- D3 unemployed: percentage of unemployed individuals within each unit of analysis
- E1 public mobility: percentage of individuals who, within the same unit of analysis, uses public transportation to go to a place of work
  (ID—Transport used to commute to the habitual place of work)
- E2 private mobility: percentage of individuals who, within the same unit of analysis, use a private vehicle to go to a place of work
  (ID—Transport used to go to the habitual place of work; habitual place of work)
- E3 walking: percentage of individuals who, within the same unit of analysis, walk to work
  (ID—Transport used to go to the habitual place of work; habitual place of work)
- F1 no cohabitants: percentage of individuals who, within the same unit of analysis, live in households where relatives or cohabitants are absent
  (ID—Marital status)
- F2 no partner: percentage of individuals who, within the same unit of analysis, live in household where the partner is absent
- G1 ownership: percentage of the individuals who, within the same unit of analysis, are owners of the dwelling
  (ID—Tenure status)
- G2 social housing: percentage of social housing from the total housing within the same unit of analysis
  (ID—Owner of the dwelling: state-owned)

## Footnotes

1 http://factominer.free.fr/.

2 https://www.xlstat.com/en/.

3 http://www.istat.it/it/censimento-popolazione/censimento-popolazione-2001.

4 https://www.comune.roma.it/web-resources/cms/documents/ElencoSuddivisioniToponomastiche_rg_A.pdf.

5 The neighbourhoods Tor di valle, Magliana vecchia and Polline Martignano have been excluded from the analysis because census data are available for a very small number of individuals (less than 10), and their indicator values always belong to the top or lower 1st percentile.

6 The black areas represents the neighbourhoods excluded from the analysis (see footnote 3 in Sect. 5).
